# Predictive Value of Assessing Diastolic Strain Rate on Survival in Cardiac Amyloidosis Patients with Preserved Ejection Fraction

**DOI:** 10.1371/journal.pone.0115910

**Published:** 2014-12-26

**Authors:** Dan Liu, Kai Hu, Stefan Störk, Sebastian Herrmann, Bastian Kramer, Maja Cikes, Philipp Daniel Gaudron, Stefan Knop, Georg Ertl, Bart Bijnens, Frank Weidemann

**Affiliations:** 1 Department of Internal Medicine I, University Hospital Würzburg, Würzburg, Germany; 2 Comprehensive Heart Failure Center, University Hospital Würzburg, Würzburg, Germany; 3 Department for Cardiovascular Diseases, University Hospital Center Zagreb and School of Medicine, University of Zagreb, Zagreb, Croatia; 4 Department of Internal Medicine II, University Hospital Würzburg, Würzburg, Germany; 5 ICREA - Universitat Pompeu Fabra, Barcelona, Spain; 6 Department of Cardiovascular Diseases, K.U. Leuven, Leuven, Belgium; University of Buenos Aires, Faculty of Medicine. Cardiovascular Pathophysiology Institute, Argentina

## Abstract

**Objectives:**

Since diastolic abnormalities are typical findings of cardiac amyloidosis (CA), we hypothesized that speckle-tracking-imaging (STI) derived longitudinal early diastolic strain rate (LSR_dias_) could predict outcome in CA patients with preserved left ventricular ejection fraction (LVEF >50%).

**Background:**

Diastolic abnormalities including altered early filling are typical findings and are related to outcome in CA patients. Reduced longitudinal systolic strain (LS_sys_) assessed by STI predicts increased mortality in CA patients. It remains unknown if LSR_dias_ also related to outcome in these patients.

**Methods:**

Conventional echocardiography and STI were performed in 41 CA patients with preserved LVEF (25 male; mean age 65±9 years). Global and segmental LS_sys_ and LSR_dias_ were obtained in six LV segments from apical 4-chamber views.

**Results:**

Nineteen (46%) out of 41 CA patients died during a median of 16 months (quartiles 5–35 months) follow-up. Baseline mitral annular plane systolic excursion (MAPSE, 6±2 vs. 8±3 mm), global LSR_dias_ and basal-septal LSR_dias_ were significantly lower in non-survivors than in survivors (all p<0.05). NYHA class, number of non-cardiac organs involved, MAPSE, mid-septal LS_sys_, global LSR_dias_, basal-septal LSR_dias_ and E/LSR_dias_ were the univariable predictors of all-cause death. Multivariable analysis showed that number of non-cardiac organs involved (hazard ratio [HR]  = 1.96, 95% confidence interval [CI] 1.17–3.26, *P* = 0.010), global LSR_dias_ (HR = 7.30, 95% CI 2.08–25.65, *P* = 0.002), and E/LSR_dias_ (HR = 2.98, 95% CI 1.54–5.79, *P* = 0.001) remained independently predictive of increased mortality risk. The prognostic performance of global LSR_dias_ was optimal at a cutoff value of 0.85 S^−1^ (sensitivity 68%, specificity 67%). Global LSR_dias_ <0.85 S^−1^ predicted a 4-fold increased mortality in CA patients with preserved LVEF.

**Conclusions:**

STI-derived early diastolic strain rate is a powerful independent predictor of survival in CA patients with preserved LVEF.

## Introduction

Systemic amyloidosis is an uncommon multisystem disease caused by the deposition of insoluble proteins in various tissues and organs. Patients with primary light-chain (AL) amyloidosis have a very poor prognosis with a median survival of 13 months from diagnosis [1]. Cardiac involvement, termed cardiac amyloidosis (CA), is observed in about 50% of patients with systemic amyloidosis, and the major driver of mortality in patients with AL amyloidosis [2], [3].

The optimal diagnostic workup for patients with suspected CA includes a combination of medical history, cardiac imaging (echocardiography, cardiac magnetic resonance imaging), electrocardiography, and histopathological examination (endomyocardial biopsy). Echocardiography is routinely used to detect cardiac abnormalities in suspected CA. The echocardiographic features of advanced CA include concentric left ventricular (LV) and right ventricular (RV) wall thickening, biatrial enlargement, granular sparkling pattern of myocardium, and diastolic dysfunction [4]–[6]. Detection of subclinical myocardial dysfunction in CA is crucial for improving therapy efficiency and predicting prognosis [7], [8]. Further insights into CA can be gained by speckle tracking derived strain rate imaging (STI), which provides more detailed information on regional myocardial deformation than conventional echocardiography. Recent studies demonstrated that longitudinal systolic dysfunction detected by STI was a typical feature of CA [9]–[12] and contributed to risk stratification in CA [11].

Diastolic abnormalities are generally recognized as the earliest manifestation of CA [13], [14] and Doppler-derived LV diastolic filling variables could predict cardiac mortality risk in CA patients [15]. A newly published study reported that early diastolic strain rate, a novel marker related to LV filling pressure, was associated with heart failure and prognosis in acute myocardial infarction patients [16]. The prognostic value of diastolic strain rate patterns in patients with CA is still unknown. The purpose of this study was to explore the predictive value of STI-derived longitudinal early diastolic strain rate (LSR_dias_) on mortality risk in CA patients with preserved left ventricular ejection fraction (LVEF).

## Methods

### Ethics Statement

Written informed consent was obtained from all patients or their guardians. The study was approved by Local Ethics Committee at the University of Würzburg and conducted in accordance to the Declaration of Helsinki.

### Study population

Forty-one patients with biopsy-proven systemic AL amyloidosis and typical echocardiographic features of cardiac involvement [11] referred to University Hospital of Würzburg were included retrospectively. For inclusion, at least one biopsy specimen from endomyocardial tissue, bone marrow, rectum, kidney, or subcutaneous fat had to be positive for Congo red staining visualized amyloid. Non-cardiac, organ involvement was defined according to the guidelines of AL [17]. Patients with coronary artery disease, more than mild concomitant valvular disease, moderate to severe arterial hypertension, and hypertrophic cardiomyopathies were excluded. Written informed consent was obtained from all patients or their guardians. The study was approved by Local Ethics Committee at the University of Würzburg and conducted in accordance to the Declaration of Helsinki.

### Electrocardiography

A standard 12-lead electrocardiography was recorded. Low QRS voltage was defined as peak to peak QRS amplitudes in each limb lead of less than 0.5 mV, and less than 1.0 mV in any precordial lead [18]. A pseudoinfarct pattern was defined as a QS wave pattern in 2 contiguous leads in the absence of previous myocardial infarction.

### Echocardiography

A standard transthoracic echocardiography was performed (GE, Vingmed Vivid 7, Horten, Norway). All measurements were performed offline in a remote workstation (EchoPAC version 112, GE, Horten, Norway) by a single experienced operator (DL). Standard two-dimensional (2D) images and Doppler recordings were obtained according to guidelines [19]. LV end-diastolic (LVEDD), end-systolic dimensions (LVESD), end-diastolic thickness of the posterior wall (LVPWd) and the septum (IVSd), LV stroke volume (Teich) and fractional shortening (Teich) were measured using standard M-mode in parasternal LV long axis views. From the apical 4-chamber view, RV end-diastolic dimension and end-systolic right atrial area were measured. RV free wall basal thickness was measured at end-diastole by M-mode or 2D echocardiography from the subcostal view. Left atrial end-systolic diameter was measured in 2D mode from the parasternal long-axis view. LVEF was measured with the biplane Simpson method in apical 4- and 2-chamber views. Average mitral annular plane systolic excursion (MAPSE) measured at the septal and lateral sites as well as tricuspid plane annular systolic excursion were obtained by M-mode in an apical 4-chamber view. LV mass, indexed to body surface area, (LVMI) was estimated by LV cavity dimension and wall thickness at end diastole [19]: LV mass (g) = 0.8×[1.04×(LVEDD + LVPWd + IVSd)^3^ – LVEDD^3^)] +0.6.

Pulsed-wave Doppler was performed in the apical 4-chamber view to obtain mitral inflow velocities for LV filling pattern evaluation. Peak velocity of early (E) and atrial (A) diastolic filling and deceleration time of E wave (DT) and isovolumic relaxation time (IVRT) were measured as well as the E/A ratio was calculated. Tissue Doppler early diastolic mitral annular velocity (E′) was acquired at the septal annular site. Diastolic function was graded according to recent guidelines [20] and not graded in patients with atrial fibrillation (n = 6).

### STI-derived systolic and diastolic deformation

**Figure 1:**
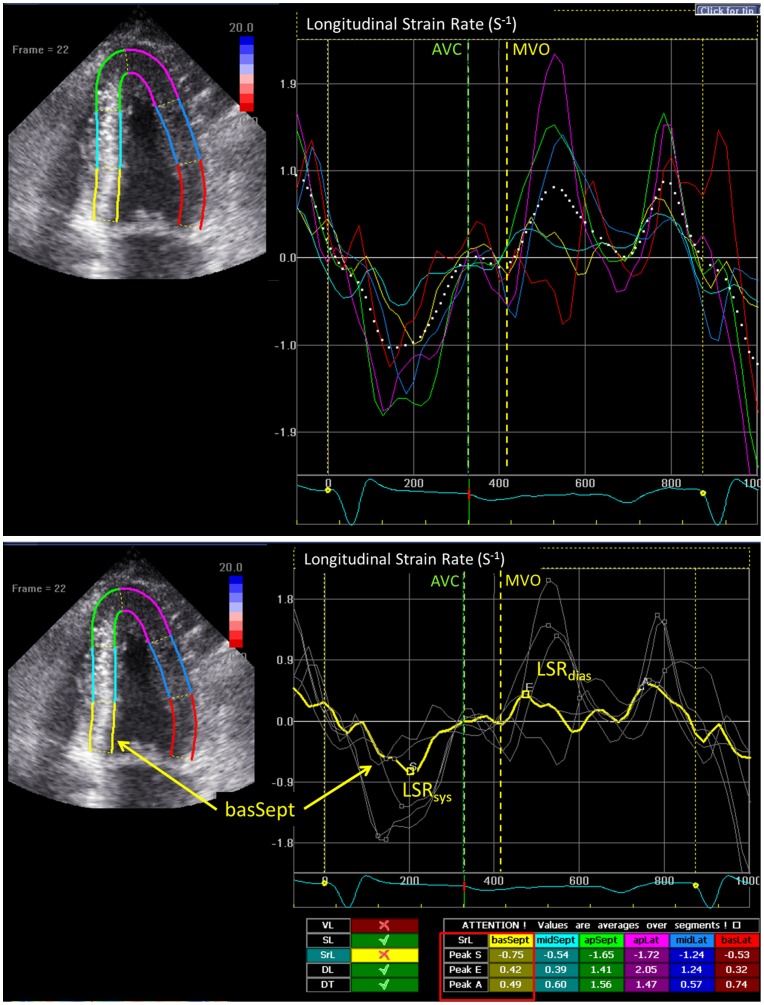
Examples for the measurement of longitudinal peak early diastolic strain rate (LSR_dias_) from two-dimensional speckle tracking imaging. On the upper panel, solid colored lines indicate corresponding segmental strain rate curves and white dashed line indicates global strain rate curve. The measurements of longitudinal systolic strain rate (LSR_sys_) and LSR_dias_ in the basal-septal segment are shown (yellow line) on the lower panel. AVC: aortic valve closure; MVO: mitral valve opening.

STI was analyzed off-line using EchoPAC software (version 112, GE, Horten, Norway). All 2D grey scale images of standard apical 4-chamber view were recorded with a frame rate of 50 to 80 frames per second. A region of interest was created by manually outlining the endocardial border at end-systolic frame on the apical 4-chamber view. The system automatically tracked the tissue within the region and divided the myocardium into six segments. Longitudinal strain rate and strain curves were obtained, and longitudinal peak systolic (LSR_sys_) and early diastolic (LSR_dias_) strain rate as well as peak systolic strain (LS_sys_) were measured in the basal, mid, and apical segments of septal and lateral walls. Global LSR_sys_, LSR_dias_ and LS_sys_ of all six segments were calculated (Fig. 1). The E/LSR_dias_ ratio was calculated as the E velocity divided by the global LSR_dias_.

Reproducibility of LSR_sys_, LS_sys_, and LSR_dias_ was assessed by repeated measurements in the same recordings. Intra-observer variation was assessed by repeated analysis of 20 randomly selected subjects and blinded to the initial results by one investigator (DL). Inter-observer variation was done on the same datasets by two observers (DL and KH). The intra- and inter-observer variability was assessed by Bland and Altman analysis and intraclass correlation coefficient.

### Primary end point

All patients were followed by clinical visit or telephone interview. The primary end point was all-cause death.

### Statistical analysis

Continuous variables were presented as mean ± standard deviation (SD) or median (quartiles) and categorical variables as percentages. Non-normally distributed variables were normalized prior to analysis using a natural logarithm or inverted values. Differences on continuous data between two groups were compared using a Student *t*-test after normalization if indicated. Categorical data were compared across groups using a Chi-square test or Fisher's exact test, as appropriate.

The optimal cut-off values of deformation variables were derived from receiver operating characteristic (ROC) analysis by maximizing the sum of the sensitivity and specificity. Survival curves were calculated by the Kaplan-Meier method, and compared by log-rank tests. Hazard ratios (HR) with 95% confidence intervals (CI) were calculated using univariable and multivariable Cox proportional-hazards regression analysis. The variables with P<0.05 in the univariable analysis were included in the multivariable models. Independent prognostic factors were identified by the multivariable Cox models with the backward stepwise method (likelihood ratio) adjusted for age and gender. Incremental model performance was assessed by changes in the Chi-square value for the regression models. Statistical significance was defined as *P*<0.05. Statistical analysis was performed using IBM SPSS, version 21 for Windows (SPSS).

## Results

### Baseline clinical and standard echocardiographic characteristics

Baseline clinical characteristics and electrocardiographic findings were similar between survivors and non-survivors except that the number of involved organs was significantly higher in non-survivors than in survivors (*P* = 0.017, table 1) while average MAPSE was significantly lower in non-survivors compared to survivors (*P* = 0.041, table 2).

**Table 1:** Clinical characteristics and electrocardiographic data.

	CA with preserved EF	Survivors	Non-survivors	*P* value
	n = 41	n = 22	n = 19	
Age (years)	65±9	65±8	65±11	0.825
Male (n; %)	61%	59%	63%	0.790
BMI (kg/m^2^)	24±3	23±2	24±4	0.355
Systolic blood pressure (mmHg)	119±19	122±17	116±21	0.354
Diastolic blood pressure (mmHg)	72±13	75±12	69±14	0.203
Heart rate (beats/min)	76±9	75±12	77±9	0.419
NYHA class	2.3±0.8	2.1±0.8	2.4±0.8	0.195
NYHA class III/IV, n (%)	44	33%	55%	0.162
Pleural effusion (%)	43%	37%	50%	0.419
Number of non-cardiac organs involved	1.5±0.9	1.2±0.8	1.8±0.8	0.017
Renal (%)	59%	45%	74%	0.069
Hepatic/gastrointestinal (%)	69%	65%	74%	0.557
Lung (%)	8%	0%	16%	0.106
Neuropathic (%)	5%	5%	5%	1.000
Soft tissues/bone (%)	10%	5%	16%	0.342
***Biomarkers***				
NT-proBNP (pg/mL)	3813 (1546–14564)	1830 (998–21882)	4543 (2846–15193)	0.352
Creatinine (mg/mL)	1.2 (0.8–1.8)	1.1 (0.7–1.9)	1.2 (0.9–1.8)	0.845
GGT (UL)	91 (32–221)	74 (36–226)	111 (29–242)	0.684
AKP (UL)	83 (67–141)	73 (62–95)	90 (68–216)	0.125
Albumin (g/dL)	3.7 (3.0–4.1)	3.7(3.4–4.0)	3.6 (2.8–4.3)	0.756
Free Kappa light chain (mg/L)	21 (6–114)	21 (17–129)	16 (1.5–114)	0.384
Free Lambda light chain (mg/L)	22 (10–122)	12 (9–122)	31 (21–327)	0.082
Kappa/Lambda ratio	1.11 (0.07–8.40)	1.43 (0.327–12.70)	0.45 (0.01–7.84)	0.343
***Cardiac-related drug therapy***				
Beta blocker	43%	50%	35%	0.368
Angiotensin converting enzyme inhibitor	38%	45%	29%	0.330
Diuretics	62%	50%	77%	0.098
***Electrocardiography***				
Unexplained low voltage	50%	50%	50%	1.000
QRS-T wave pseudo-infarct changes	44%	45%	42%	0.855
I/II° atrioventricular block	64%	53%	75%	0.188
Left/right bundle branch block	39%	50%	26%	0.129

Non-survivors vs. survivors *P*<0.05 indicated significantly different. CA: cardiac amyloidosis; BMI: body mass index; NYHA class: New York Heart Association functional classification; NT-proBNP: N-terminal pro-B-type natriuretic peptide; GGT: Gamma-glutamyl transpeptidase; AKP: alkaline phosphatase enzyme.

**Table 2:** Echocardiographic characteristics.

	CA with preserved EF	Survivors	Non-survivors	*P* value
	n = 41	n = 22	n = 19	
***Cardiac size***				
LV end-diastolic dimension (mm)	43±6	42±6	43±7	0.625
LV end-systolic dimension (mm)	29±5	29±5	29±6	0.802
IVS thickness (mm)	15±3	14±3	15±3	0.557
LV posterior wall thickness (mm)	14±3	14±3	15±2	0.333
RV dimension (mm)	34±5	33±5	36±5	0.104
RV lateral wall thickness (mm)	6±1	6±1	6±1	0.647
Relative wall thickness	0.68±0.17	0.67±0.18	0.70±0.17	0.542
LA diameter (mm)	42±7	41±7	43±6	0.350
RA area (cm^2^)	18±5	17±5	20±5	0.140
LVMI (g/m^2^)	138±47	130±47	145±47	0.304
***LV/RV Systolic function***				
LV fractional shortening (%)	31±7	31±7	30±7	0.652
LV EF (%)	62±6	62±6	61±6	0.872
Stroke volume (ml)	46±17	42±18	50±14	0.140
MAPSE (mm)	7±3	8±3	6±2	0.041
TAPSE (mm)	15±4	16±5	14±4	0.203
***LV diastolic function***				
E wave (m/s)	0.87±0.24	0.86±0.26	0.88±0.22	0.841
E/A	1.46±0.88	1.31±0.81	1.62±0.95	0.307
E/E′	20±9	19±8	21±10	0.456
DT (ms)	176±62	184±67	167±57	0.377
IVRT (ms)	89±19	89±18	88±20	0.844
Diastolic filling pattern:				
Normal/abnormal relaxation/pseudonormal/restrictive/atrial fibrillation	1/17/9/8/6	1/10/4/3/4	0/7/5/5/2	0.549
***SPAP (mmHg)***	38±15	36±14	39±16	0.507
***Pericardial effusion***	49%	50%	47%	0.867
***Myocardial sparkling texture***	85%	86%	84%	1.000

Non-survivors vs. survivors *P*<0.05 indicated significantly different. LV: left ventricle; RV: right ventricle; LA: left atrium; RA: right atrium; IVS: interventricular septum; LVMI: LV mass indexed to body surface area; EF: ejection fraction; MAPSE: average of mitral annular plane systolic excursion measured at the septal and lateral sites; TAPSE: tricuspid annular plane systolic excursion; E: early diastolic peak filling velocity; A: late diastolic peak filling velocity; E′: tissue Doppler early diastolic septal mitral annular velocity. DT: deceleration time of early diastolic peak velocity; IVRT: isovolumic relaxation time; SPAP: systolic pulmonary artery pressure.

### STI-derived systolic and diastolic deformation characteristics

Among systolic deformation parameters (table 3), mid-septal LS_sys_ tended to be lower in non-survivors than survivors (*P* = 0.052). The typical longitudinal apex-to-base strain gradient (septal apical to basal longitudinal systolic strain ratio greater than 2.1) was evidenced in 67.5% CA patients but did not discern between survivors and non-survivors (62% vs. 74%, *P* = 0.427). Diastolic strain rate analysis showed that global LSR_dias_ and basal-septal LSR_dias_ were significantly lower in non-survivors than in survivors (both *P*<0.05).

**Table 3:** Longitudinal systolic and diastolic strain rate and strain.

	Survivors	Non-survivors	P value
	n = 22	n = 19	
Global LS_sys_ (%)	−13±4	−11±3	0.143
Global LSR_sys_ (S^−1^)	−0.8±0.3	−0.8±0.2	0.603
Global LSR_dias_ (S^−1^)	0.97±0.30	0.76±0.26	0.024
E/LSR_dias_	0.97±0.33	1.36±0.84	0.057
Segmental LS_sys_ (%)			
Apical septal	−19±6	−17±6	0.339
Mid septal	−12±5	−9±4	0.052
Basal septal	−8±5	−6±3	0.086
Apical lateral	−17±5	−17±6	0.638
Mid lateral	−11±4	−10±4	0.398
Basal lateral	−8±5	−7±4	0.383
Septal LSsys_api/bas_	3.0±2.0	3.5±1.7	0.447
Segmental LSR_sys_ (S^−1^)			
Apical septal	−1.3±0.5	−1.4±0.5	0.533
Mid septal	−0.7±0.3	−0.6±0.3	0.209
Basal septal	−0.5±0.3	−0.4±0.2	0.223
Apical lateral	−1.3±0.5	−1.3±0.3	0.848
Mid lateral	−0.9±0.4	−0.8±0.2	0.219
Basal lateral	−0.8±0.4	−0.6±0.2	0.228
Segmental LSR_dias_ (S^−1^)			
Apical septal	1.6±0.6	1.5±0.6	0.601
Mid septal	0.8±0.3	0.6±0.3	0.103
Basal septal	0.6±0.4	0.4±0.2	0.015
Apical lateral	1.6±0.7	1.6±0.6	0.974
Mid lateral	1.1±0.5	0.9±0.4	0.269
Basal lateral	0.9±0.6	0.6±0.5	0.056

Non-survivors vs. survivors *P*<0.05 indicated significantly different. LS_sys_: longitudinal peak systolic strain; LSR_sys_: longitudinal peak systolic strain rate; LSR_dias_: longitudinal peak early diastolic strain rate; E/LSR_dias_: early diastolic peak filling velocity to global LSR_dias_ ratio; LSsys_api/bas_: septal apical to basal longitudinal systolic strain ratio.

### Reproducibility

Systolic and diastolic deformation parameters in 120 segments were measured for the intra- and inter-observer variability. Mean differences ±2 SD for LSR_sys_, LS_sys_, and LSR_dias_ were 0.04±0.48 S^−1^, 0.66±5.48%, and −0.01±0.58 S^−1^ for intra-observer agreement and 0.02±0.52 S^−1^, 0.36±5.64%, and 0.04±0.68 S^−1^ for inter-observer agreement. Intraclass correlation coefficient for LSR_sys_, LS_sys_, and LSR_dias_ were 0.877 (95% CI 0.831–0.911), 0.907 (95% CI 0.871–0.933), and 0.913 (95% CI 0.880–0.938) for intra-observer reliability and 0.854 (95% CI 0.800–0.894), 0.903 (95% CI 0.865–0.930), and 0.887 (95% CI 0.844–0.918) for inter-observer reliability.

### Predictive value of systolic and diastolic strain rate for survival

Nineteen patients (46%) died during a median follow-up time of 16 (5–35) months. As shown in table 4, univariable predictors of all-cause mortality included NYHA functional class, number of non-cardiac organ involved, MAPSE, mid-septal LS_sys_, global LSR_dias_, basal-septal LSR_dias_ and E/LSR_dias_ (all *P*<0.05). Table 5 showed the correlations among the deformation parameters, suggesting that E/LSR_dias_ was closely related to global LSR_dias_ (R = −0.712). Therefore, E/LSR_dias_ and global LSR_dias_ were respectively added into two multivariable models (table 6, Model A and B). Multivariable analysis showed that number of non-cardiac organs involved (HR = 1.96, 95% CI 1.17–3.26, *P* = 0.010), global LSR_dias_ (HR = 7.30, 95% CI 2.08–25.65, *P* = 0.002), and E/LSR_dias_ (HR = 2.98, 95% CI 1.54–5.79, *P* = 0.001) remained independently predictive of increased mortality risk (table 6).

**Table 4:** Prediction for Mortality by univariable Cox proportional hazard regression analysis.

	Hazard ratio (95% CI)	*P* value
Age (years)	0.98 (0.93–1.02)	0.338
Male	0.80 (0.31–2.04)	0.638
NYHA class	1.77 (1.02–3.09)	0.044
Number of non-cardiac involved organs	2.00 (1.15–3.46)	0.014
LVMI (g/m^2^)	1.00 (0.99–1.01)	0.401
LAD (mm)	1.03 (0.97–1.10)	0.331
MAPSE (mm)	1.24 (1.02–1.50)	0.034
DT (ms)	0.99 (0.98–1.00)	0.069
E/E′	1.05 (0.99–1.10)	0.091
Global LS_sys_ (%)	2.30 (0.88–6.03)	0.097
Septal LSsys_api/bas_	1.09 (0.91–1.31)	0.339
Mid-septal LS_sys_ (%)	1.10 (1.01–1.22)	0.027
Global LSR_dias_ (S^−1^)	4.90 (1.73–13.89)	0.003
Basal-septal LSR_dias_ (S^−1^)	3.14 (1.53–6.45)	0.002
E/LSR_dias_	3.27 (1.70–6.29)	<0.001

CI: confidence interval. For abbreviations, see table 2 and 3.

**Table 5:** Spearman's Correlations among deformation parameters.

	E/LSR_dias_	Mid-septal LS_sys_	Basal-septal LSR_dias_	Global LSR_dias_
E/LSR_dias_	1	0.576 (*P*<0.001)	−0.358 (*P* = 0.023)	−0.721 (*P*<0.001)
Mid-septal LS_sys_	0.576 (*P*<0.001)	1	−0.535 (*P*<0.001)	−0.503 (*P* = 0.001)
Basal-septal LSR_dias_	−0.358 (*P* = 0.023)	−0.535 (*P*<0.001)	1	0.292 (*P* = 0.068)
Global LSR_dias_	−0.721 (*P*<0.001)	−0.445 (*P* = 0.004)	0.292(*P* = 0.068)	1

**Table 6:** Multivariable Cox proportional hazard regression analysis.

Model A	Hazard ratio (95% CI)	*P* value	Model B	Hazard ratio (95% CI)	*P* value
NYHA class	1.56 (0.89–2.74)	0.122	NYHA class	1.55 (0.89–2.71)	0.122
Number of non-cardiac organs involved	1.96 (1.17–3.26)	0.010	Number of non-cardiac organs involved	1.72 (1.00–2.99)	0.052
MAPSE (mm)	1.15 (0.96–1.38)	0.137	MAPSE (mm)	1.15 (0.96–1.38)	0.137
Mid-septal LS_sys_ (%)	1.00 (0.87–1.14)	0.946	Mid-septal LS_sys_ (%)	1.01 (0.89–1.14)	0.878
Basal-septal LSR_dias_ (S^−1^)	1.88 (0.78–4.52)	0.158	Basal-septal LSR_dias_ (S^−1^)	1.72 (0.60–4.97)	0.311
Global LSR_dias_ (S^−1^)	7.30 (2.08–25.65)	0.002	E/LSR_dias_	2.98 (1.54–5.79)	0.001

Adjusted for age and gender with a backward stepwise method (likelihood ratio). For abbreviations, see table 2 and 3.

**Figure 2:**
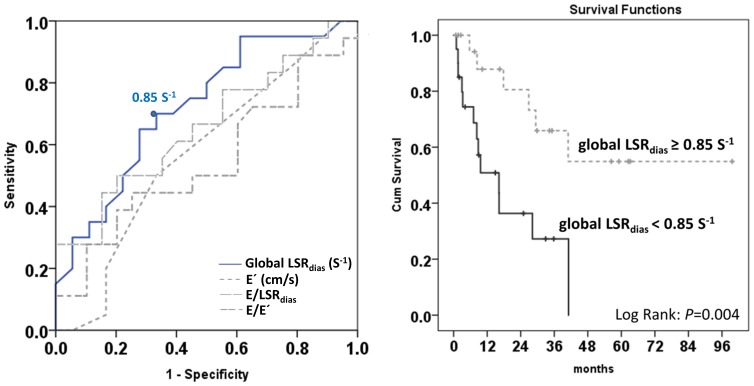
The receiver operating characteristic (ROC) analysis of global early diastolic strain rate (LSR_dias_) for predicting mortality (left) and cumulative survival stratified by the optimal cut-off value for global LSR_dias_ (right). Global LSR_dias_ serves as the best marker for predicting mortality in cardiac amyloidosis patients with preserved ejection fraction (area under of ROC curve: 0.72 (0.56–0.89), *P* = 0.019). CA Patients with global LSR_dias_ <0.85 S^−1^ suggests about 4-fold increase of all-cause mortality than those with preserved global LSR_dias_ value.

ROC analysis showed global LSR_dias_ outperformed conventional diastolic parameters (E′ and E/E′) as well as E/LSR_dias_ for predicting mortality in this cohort (Fig. 2 left). The prognostic performance of global LSR_dias_ was optimal at a cutoff value of 0.85 S^−1^ (sensitivity 68%, specificity 67%, positive predictive value 65%, and negative predictive value 70%). CA Patients with global LSR_dias_ <0.85 S^−1^ was related with a 4-fold increase of all-cause mortality than those with global LSR_dias_ ≥0.85 S^−1^ (Fig. 2 right).

### Incremental predictive power of STI information

**Figure 3:**
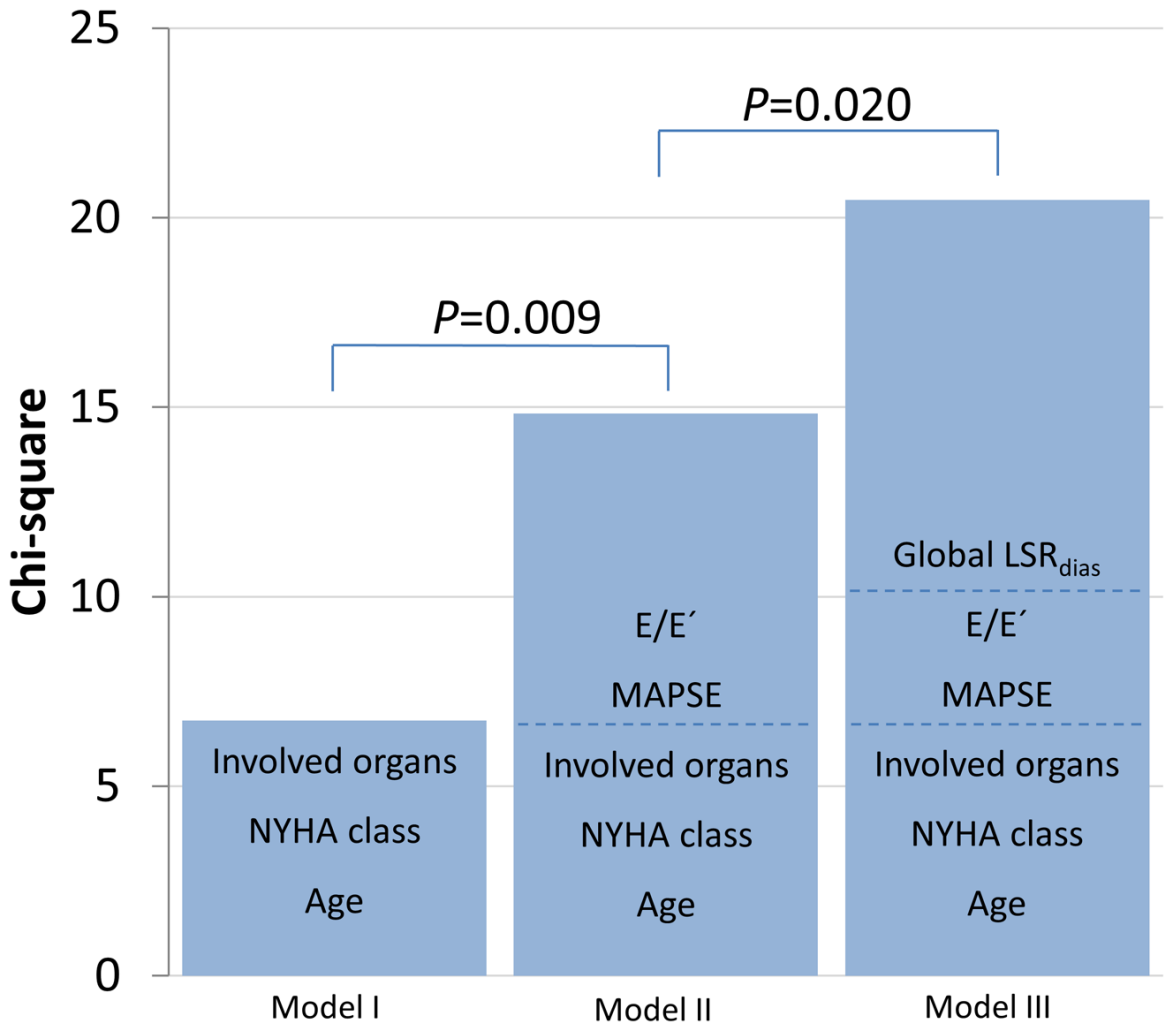
Incremental model performance for predicting prognosis assessed by starting with the clinical variables (Model I: age, NYHA class, and number of non-cardiac organs involved), followed by the conventional echocardiographic parameters (Model II: adding MAPSE and E/E′ to model I), and finally by adding advanced global LSR_dias_ (Model III). MAPSE: mitral annular plane systolic excursion; E/E′: early diastolic peak filling velocity to tissue Doppler early diastolic septal mitral annular velocity ratio. LSR_dias_: longitudinal peak early diastolic strain rate.

Clinical variables (Model I) including age, NYHA class and number of involved organs were entered in the first step of a multivariable Cox model are predictive of mortality (Chi-square 6.74, *P* = 0.012). In Model II, adding conventional echocardiographic parameters (MAPSE and E/E′) to Model I enhanced the explanatory power (Chi-square 14.83, *P* = 0.009 vs. Model I). Adding global LSR_dias_ (Model III) further improved the prognostic performance (Chi-square 20.47, *P* = 0.020 vs. Model II; Fig. 3).

## Discussion

This is the first attempt to explore the prognostic value of STI-derived global LSR_dias_ for predicting overall mortality risk in CA patients with preserved LVEF. We found that global LSR_dias_ was superior to conventional diastolic parameters (E′ and E/E′) for predicting outcome and improved the risk stratification in CA patients with preserved LVEF(>50%). Reduced global LSR_dias_ (<0.85 S^−1^) independently predicted a 4-fold increased mortality in these patients, while mid-septal LS_sys_ only showed borderline significance (*P* = 0.052) on outcome. Adding global LSR_dias_ to clinical data and conventional echocardiographic variables offered incremental prognostic value.

### Systolic and diastolic functions and outcome in CA patients with preserved LVEF

It was shown that LV wall thickening and reduced LVEF are associated with higher mortality risk in CA patients [2]. In the present study, we compared the prognostic values of traditional and advanced echocardiographic parameters in a small subset of CA patients with preserved systolic function and similar LV wall thickness. Our results showed that, MAPSE, a simple classic parameter reflecting longitudinal LV function [21], was significantly lower in non-survivor group, suggesting longitudinal dysfunction was associated with poor outcome in CA patients with preserved LVEF. Recent studies reported the diagnostic and prognostic value of two- or three-dimensional STI derived strain and strain rate in various cardiovascular diseases [22], [23]. Bellavia et al. demonstrated that reduced global and basal longitudinal systolic strains were independently linked with increased all-cause mortality in AL amyloidosis patients [24], [25]. We, and other groups, recently also showed that an intra-wall longitudinal systolic strain gradient with preserved LS_sys_ at apical segments and significantly reduced LS_sys_ at mid and basal segments is a typical strain pattern for CA [9]–[11]. In the current cohort, reduction in longitudinal systolic strain at mid segments of the septum (mid-septal LS_sys_) was evidenced and associated with increased mortality. But MAPSE and mid-septal LS_sys_ are only univariable but not independent predictors of all-cause mortality. The reason for the non-independent predictive value of MAPSE and mid-septal LS_sys_ might originate from the fact that LVEF was preserved and by nature related to a better systolic function in our cohort.

Diastolic abnormalities are typical features of CA and related to the degree of amyloid infiltration in heart, LV compliance becomes increasingly reduced resulting in a “stiff ventricle” with increasing myocardial amyloid infiltration [13], [14]. Early CA typically presents with a mildly increased wall thickness and an abnormal relaxation filling pattern, while advanced CA shows a grossly increased wall thickness and a restrictive filling pattern with markedly shortened deceleration time and a low velocity A wave [26], [27]. Pulsed tissue Doppler (E′) was useful to detect early myocardial diastolic impairment, and the E/E′ ratio was related to increased LV filling pressure [28]. Consistent with these findings, 34 out of 35 (97%) CA patients with sinus rhythm showed various degrees of diastolic filling abnormalities (relaxation abnormal 48%, pseudonormal 23%, restrictive 17%) and 72% patients had E/E′>15 in our cohort. Wang et al. demonstrated that global strain rate during the isovolumetric relaxation period (SR_IVR_) derived by STI is strongly related to hemodynamic indices of LV relaxation both in an animal model and in patients, and E/SR_IVR_ can predict LV filling pressures, particularly in patients with normal ejection fraction [29]. However, Kasner et al. revealed only a weak correlation of SR_IVR_ with LV stiffness coefficient in heart failure patients with normal ejection fraction by a simultaneous comparison during invasive conductance catheterization [30]. In our study, global SR_IVR_ was not quantified as with speckle tracking imaging the temporal resolution is not good enough for the detection of change of deformation during this short period. Thus, only global and segmental strain rate during early LV filling were assessed. The study by Wang et al. also reported that strain rate during early LV filling (i.e. SR_dias_) was negatively correlated to tau (r = −0.56) and pulmonary capillary wedge pressure (r = −0.46) in humans, although this association was weaker than that of SR_IVR_ [29]. Kasner et al. showed that despite there was a reasonable correlation between STI derived diastolic parameters (SR_dias_, SR_IVR_, E/SR_dias_, E/SR_IVR_) and LV relaxation, strain rate imaging is not superior in diagnosing diastolic dysfunction in patients with preserved EF as compared with established tissue Doppler parameter E/E′ [30]. Different from aforementioned studies, present study focused on the relationship of early diastolic strain rate with the outcome of CA patients. Our data showed that global LSR_dias_ and E/LSR_dias_ remained independent predictor of mortality in these patients. Moreover, ROC analysis showed global LSR_dias_ outperformed conventional diastolic parameters (E′, E/E′, and E/LSR_dias_) for predicting mortality in this cohort. In a previous study, Kim et al. reported that LSR_dias_ was related with LV geometric remodeling patterns in hypertensive subjects, and the lowest LSR_dias_ value was evidenced in patients with concentric hypertrophy [31]. It is therefore not surprising that significantly reduced LSR_dias_ was detected in CA patients with preserved LVEF since concentric hypertrophy is one typical pathological feature of these patients. Our study extends previous findings by demonstrating that LSR_dias_ is not only reduced but also a prognostic marker for poor outcome in CA patients with preserved EF, superior to other diastolic parameters.

### Incremental predictive power of STI information

When focusing on prognosis in CA, three different aspects should be evaluated: 1) Clinical symptoms, 2) general organ involvement, and 3) cardiac stage of disease progression. In our cohort, NYHA class and number of non-cardiac organs involved were related to increased mortality.

Kristen et al. found that NYHA class was a risk factor but number of organs involved (including heart) was not associated with increased mortality in AL patients [32]. The underlying reason might be associated with the patient selection, every patient had cardiac involvement because of the inclusion criteria and CA patients have a much higher mortality risk, and involvement of other non-cardiac organs seems to be an additional risk factor for death as shown in CA patients of this cohort.

Our results showed that entering clinical variables including age, NYHA class and number of involved organs in the multivariable Cox regression model are predictive of mortality (Model I, *P* = 0.012). Adding conventional echocardiographic parameters (MAPSE and E/E′) to Model I (Model II) enhanced the explanatory power (*P* = 0.009 vs. Model I). Adding global LSR_dias_ (Model III) further improved the prognostic performance (*P* = 0.020 vs. Model II). Thus, on top of clinical profile and conventional echocardiographic markers, a reduction in global LSR_dias_ serves as an independent and incremental risk predictor of all-cause death in CA patients with preserved LVEF.

### Study limitations

The sample size was relatively small in this study. Future studies with larger patient cohort are warranted to verify the finding in the present study and to compare early diastolic strain rate changes between patients with light chain amyloidosis and non-light chain amyloidosis. Recent studies showed that high sensitive cardiac troponin-T might play an important role for the assessment of prognosis in AL amyloidosis [8], [32], [33]. Due to the retrospective feature of this study, there were few data on high sensitive cardiac troponin-T in this cohort. Thus, further prospective conducted studies are necessary to analyze the additional prognostic performance of high sensitive cardiac biomarkers in CA patients with preserved LVEF.

In addition, rapid early diastolic untwist in normal hearts servers as an important component of diastolic suction, and it could be diminished in the earliest stages of diastolic dysfunction [34]. Experimental studies have shown that LV twist at end systole and at end diastole is linearly related to volume, and this relationship is independent of variations in contractility, afterload, and heart rate [35]. Previous study found that STI-derived apical diastolic untwist was significantly correlated to established echocardiographic measures of diastolic function [36]. Further investigation is warranted on the value of STI-derived LV untwisting for diagnosing diastolic dysfunction and for predicting outcome in patients with CA.

## Conclusions

Speckle tracking imaging-derived diastolic deformation improves the risk assessment of CA patients with preserved LVEF. A reduction in global LSR_dias_ serves as an independent and incremental risk predictor of all-cause death in CA patients with preserved LVEF.
